# Prevalence of Closure of Large Macular Hole with Inverted Internal Limiting Membrane Flap Technique in A Tertiary Care Hospital of Nepal

**DOI:** 10.31729/jnma.4393

**Published:** 2019-06-30

**Authors:** Kiran Shakya, Ram Prasad Pokharel, Om Krishna Malla

**Affiliations:** 1 Department of Ophthalmology, Kathmandu Medical College and Teaching Hospital, Kathmandu, Nepal

**Keywords:** *inverted ILM flap*, *large idiopathic macular hole*, *visual acuity*

## Abstract

**Introduction:**

Large macular holes cause significant reduction in central visual acuity. The aim of the study is to find out short term anatomical and visual outcomes of inverted internal limiting membrane flaps technique for large macular holes (base diameter>1000**/vm**) surgery in patients at a tertiary care hospital.

**Methods:**

A descriptive cross-sectional study was conducted in a tertiary care hospital from January 2018 to December 2018 after ethical clearance from the institutional review committee. The study was done in 12 patients with idiopathic macular holes (base diameter>1000**/vm**), they were repaired with 25 gauge pars plana vitrectomy with brilliant blue assisted large inverted internal limiting membrane flap technique. Statistical analyses were performed using SPSS 19.0

**Results:**

All twelve eyes had complete anatomical closure. Mean best corrected visual acuity preoperatively was 1.48 logMAR±0.246. The mean macular hole base diameter was 1217.0± 196.77**/vm.** The mean age of patients was 68.75±4.97 years. Postoperatively, mean best corrected visual acuity was 0.978 logMAR±0.12. There were no postoperative complications. All the patients perceived decreased size of central scotoma.

**Conclusions:**

Inverted internal limiting membrane flaps for large macular holes is suitable method for closure of the very large hole, restoration of functional vision and decreased size of central scotoma.

## INTRODUCTION

Unilateral very large idiopathic macular holes (>1000 **/vm**) are found in elderly age as the vision in the affected eye is over shadowed by cataract and optimal vision of other eye. The inverted internal limiting membrane (ILM) flap is an effective technique for anatomical closure and improved visual function in large idiopathic full thickness macular hole (more than 400 **/vm**) patients.^1^

Mahalingam et al. found 100 percent closure rate of large macular hole (mean minimal diameter 800 **/vm**) with inverted ILM flaps.^2^ In addition, par plana vitrectomy reduces vitreo-macular tangential tractions which Gass found vital causes for different stages of idiopathic macular holes.^3^

The aim of the study is to find out short term anatomical and visual outcomes of inverted internal limiting membrane (ILM) flaps technique for large macular holes (base diameter>1000 **/vm**) surgery in patients at a tertiary care hospital.

## METHODS

This is the descriptive cross-sectional study conducted at tertiary care hospital at Kathmandu from January 2018 to December 2018 and ethical clearance was obtained from the Institutional Review Committee (IRC). Data was collected continuously during the study period. Study population were the patients who underwent surgery for large idiopathic macular holes. Patients with idiopathic large macular holes (MH base diameter>1000 /vm) were included in this study. Patient with MH with base diameter<1000 /vm, high myopic, traumatic, and media opacities were excluded from the study.

Sample size was calculated using below mentioned formula and samples were collected using convenience sampling method.


$$\text{n}={\text{Z}}^{2}\times (\text{pq)/}{\text{d}}^{2}$$

where,

n = sample size
p = prevalence of macular hole closure with inverted internal limiting membrane flap technique, 95% (educated guess)
q = 1-p
d = margin of error, 13%
Z = 1.96 at 95% CI

After taking non-response rate of 10%, the total sample size was calculated to be 12.

Patients underwent recording of best-corrected visual acuity (BCVA), fundus examination, SD OCT (Ziess, cirrus) scan for measurement of macular hole base diameter at preoperative and follow up 1 month and 3 months. Each patients were asked whether they perceived decreased size of scotoma or not. Spectral Domain-Optical Coherence Tomography images were taken preoperative and postoperative 1 month and 3 months follow up to assess the anatomical outcome of surgery and best corrected visual acuity was recorded to know the functional outcome during each visit

Selection bias and information bias was minimized as possible. Statistical analyses were performed using SPSS 19.0, point estimate at 95% CI was done along with proportion and frequency of the binary data.

## RESULTS

Twelve eyes had showed 12 (100%) macular hole closure rate with inverted ILM flap technique. Mean BVCA pre-operatively was 1.48 log MAR ±SD 0.246. The mean macular hole base diameter was 1217.0 /vm (1036-1571 /vm). Post-operatively, mean BCVA was 0.978 log MAR ±SD 0.12. The mean age of patients was 68.75±4.97 years. Female were prepondent 8 (70%).

**Table 1.** Description of patients of their BCVA and SDOCT findings.

MH base diameter (µm)	Preop BCVA (logMAR)	3 months Postop BCVA (logMAR)	Postop macular hole
1036	1.3	1.0	closed
1052	1.48	1.0	closed
1058	1.3	0.77	closed
1059	1.48	1.0	Closed
1061	1.48	1.17	Closed
1158	1.77	1.0	Closed
1186	1.77	1.07	Closed
1200	1.0	0.77	Closed
1216	1.0	0.77	closed
1481	1.48	1	Closed
1526	1.77	1	Closed
1571	1.3	1	Closed

**Table 2.** Mean value of macular hole and BCVA.

MH base diameter±SD (µm)	Preop BCVA (log MAR±SD)	3 months Postop BCVA (logMAR±SD)
1217.4±196.77	1.48±0.246	0.978±0.12

## DISCUSSION

Advancement in micro instruments like vitrectomy cutter and intraocular ILM peeling forceps helped vitrectomy surgery with better outcome. Introduction of ILM staining dyes visualize the membrane more easily during ILM peeling. Among all Michalewska Z et al. firstly proved the usefulness of ILM flap to prevent the postoperative flat-open appearance of a macular hole repair and improvement of the functional outcomes of macular holes with a diameter greater than 400 /vm.^1^ Mahalingam P et al. showed similar results with inverted flap technique for repairment of minimal macular hole diameter greater than 800 /vm.^2^ As ILM is a basement membrane and part of muller cells, it induced glial cell proliferation to fill large macular holes with tissue over time. The thin rim attachment stabilized the peeled off ILM flap in the macular hole. The ILM flap induced proliferation of glial tissue to fill and secure the hole and to support the surrounding photoreceptor cells.^2,4^

In our study, twelve eyes showed 100% macular hole closure rate with inverted ILM flap technique and other studies had similar closure rate for large macular hole.^2,5^

Postoperative visual acuity was improved from 1.48 logMAR to 0.978 logMAR. Similar studies found improvement of mean visual acuity after large treated macular hole surgery with inverted ILM flaps.^5,6^ Guber J et al. showed upto 2 lines improvement in BCVA by first postoperative follow up.^7^

All the twelve patients perceived decreased the size of scotoma in the operated eye after surgery and were satisfied with postoperative functional visual improvement. Larger study group and longer follow-up period is required to further evaluate this method. Since this is a hospital based study, the findings of the study cannot be generalized to the population.

## CONCLUSIONS

There was improvement in anatomical, visual outcomes and alleviation of central scotoma for large macular hole (base diameter>1000 v m) surgery of inverted internal limiting membrane flaps technique for large macular holes (base diameter>1000/vm) surgery in patients compared to other studies.

## Conflict of Interest

**None.**
